# Islet-1 Immunoreactivity in the Developing Retina of *Xenopus laevis*

**DOI:** 10.1155/2013/740420

**Published:** 2013-11-11

**Authors:** Guadalupe Álvarez-Hernán, Ruth Bejarano-Escobar, Ruth Morona, Agustín González, Gervasio Martín-Partido, Javier Francisco-Morcillo

**Affiliations:** ^1^Departamento de Biología Celular, Facultad de Ciencias, Universidad de Extremadura, Avenida de Elvas s/n, 06071 Badajoz, Spain; ^2^Departamento de Biología Celular, Facultad de Biología, Universidad Complutense, Ciudad Universitaria, C/José Antonio Novais 12, 28040 Madrid, Spain

## Abstract

The LIM-homeodomain transcription factor Islet1 (Isl1) has been widely used as a marker of neuronal differentiation in the developing visual system of different classes of vertebrates, including mammals, birds, reptiles, and fish. In the present study, we analyzed the spatial and temporal distribution of Isl1-immunoreactive cells during *Xenopus laevis* retinal development and its relation to the formation of the retinal layers, and in combination with different markers of cell differentiation. The earliest Isl1 expression appeared at St29-30 in the cell nuclei of sparse differentiating neuroblasts located in the vitreal surface of the undifferentiated retina. At St35-36, abundant Isl1-positive cells accumulated at the vitreal surface of the neuroepithelium. As development proceeded and through the postmetamorphic juveniles, Isl1 expression was identified in subpopulations of ganglion cells and in subsets of amacrine, bipolar, and horizontal cells. These data together suggest a possible role for Isl1 in the early differentiation and maintenance of different retinal cell types, and Isl1 can serve as a specific molecular marker for the study of retinal cell specification in *X. laevis*.

## 1. Introduction

The developing retina is an attractive system for studying different aspects of cell differentiation owing to its easy accessibility and well-organized laminar structure ultimately containing six well-characterized classes of neurons. Retinogenesis in vertebrates is stereotyped in an ordered fashion: retinal ganglion cells are always born first, followed by horizontal, amacrine, and cone cells, and finally by bipolar, rod, and Müller cells [1, 2]. During retinal cell type development, transcription factors play critical roles in the generation of diverse neuronal phenotypes, and genetic manipulation of these molecules often leads to an alteration of one or more retinal cell phenotypes [3–6]. The LIM-homeodomain transcription factor Islet-1 (Isl1) orchestrates cell fate decisions in a variety of systems [7, 8]. Different studies have shown that, in the retina, Isl1 is expressed in mature and differentiating ganglion, amacrine, bipolar, and horizontal cells, suggesting that it plays a pivotal role in the maturation of these cell types in fish [9–11], reptiles [12], birds [13–21], and mammals [22–28]. In particular, Isl1 expression has been demonstrated to be required for neuronal progenitors to specify retinal ganglion cell fate in mammals, activating genes essential for cell differentiation [25, 26, 28]. In addition, it participates in the regulation of the development of cholinergic amacrine cells in mammals [22–24] and birds [29]. It also controls the differentiation of bipolar cells in mammals [22, 23]. Furthermore, Isl1 is involved in horizontal cell determination [16, 20] and regulates the morphogenesis of subsets of postmigratory horizontal cells in the chick [19]. Surprisingly, although Isl1 is not normally expressed by horizontal cells in the developing and mature mouse retina [22–24], it participates in determining horizontal cell number [30].

There have been few reports describing Isl1 expression during amphibian central nervous system development. The South African clawed frog *Xenopus laevis* (Daudin, 1802) is a suitable model to study different aspects of central nervous system development, and, recently, implications of Isl1 in diverse aspects of regional development and neuronal specification in the forebrain have been demonstrated [31]. Furthermore, several authors have used Isl1 as an early marker of ganglion cells during development in this anuran species [32–34], but the detailed spatiotemporal expression of this transcription factor during retinal development has not previously been described. The aim of our study was to analyze immunohistochemically the onset and the dynamic expression of Isl1 during retinal development of *X. laevis*. The structural arrangement of the retina was examined in toluidine blue-stained resin sections and in cryosections labeled with DAPI. We characterized the subpopulations of Isl1-expressing cells by both morphological and topographical features, but also by double immunolabeling with other retinal markers, whose distribution we have previously studied in the developing and mature retina of different vertebrates [9–12, 35, 36]. The results indicated that Isl1 is expressed at early stages of retinal development and maintained through juvenile stages, implying potential roles in retinal cell specification, differentiation, and maintenance in *X. laevis*.

## 2. Material and Methods

### 2.1. Animals and Tissue Processing

**Figure 1:**
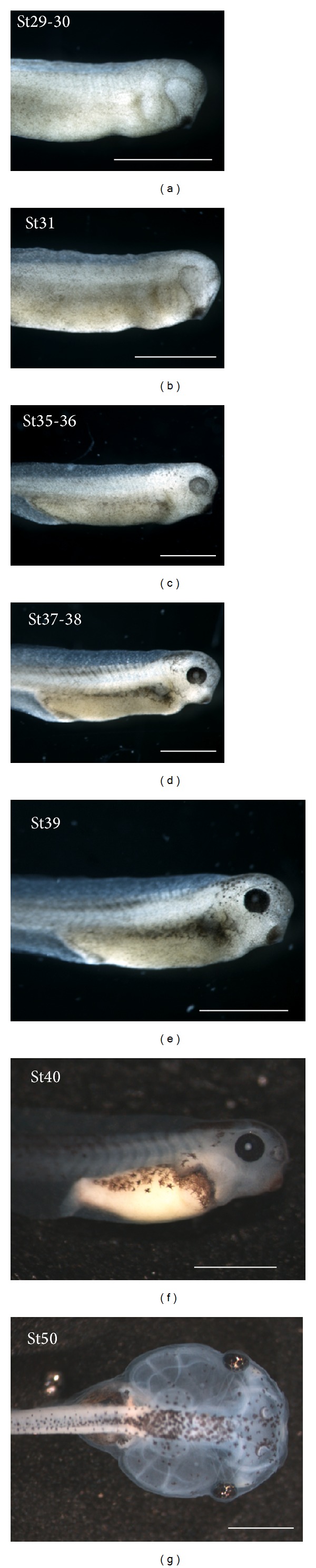
Stereomicroscope images of selected lateral views of *Xenopus laevis* tailbud embryos ((a)–(f)) and a dorsal view of a free-swimming tadpole (g) according to developmental stages (St) of Nieuwkoop and Faber [37]. Scale bars: 1 mm.

All animals were treated according to the regulations and laws of the European Union (EU Directive 2010/63/EU) and Spain (Royal Decree 53/2013) for care and handling of animals in research, after approval from the Universities of Extremadura and Complutense to conduct the experiments described. The number of animals used in the present study was the minimum to guarantee the correct interpretation of the results. A total of 82 *Xenopus laevis* embryos, larvae, and juveniles were used (Table 1). *X. laevis* embryos and larvae were carefully staged in accordance with Nieuwkoop and Faber [37]. Representative developmental stages are shown in Figure 1. Adult *X. laevis* were purchased from commercial suppliers (XenopusOne, Dexter, MI, USA) and the different developing specimens were obtained by breeding induced by chorionic gonadotropin (Pregnyl; Organon, West Orange, NJ, USA) and kept in tap water at 20–25°C. Young larvae were raised on Mikropan Growth Food (Sera, Heinsberg, Germany), and older larvae and juveniles were fed liver meat. At appropriate times, embryos, larvae, and juveniles were deeply anesthetized by immersion in a 0.3% solution of tricaine methanesulfonate (MS222, pH 7.4; Sigma Chemical, St. Louis, MO, USA) and used for the different sets of experiments. Specimens were fixed by immersion for 20 hours at 4°C in 4% paraformaldehyde (PFA) in PB (phosphate-buffered solution 0.1 M, pH 7.4) or MEMFA (0.1 M MOPS—4-morpholinopropano sulfonic acid—2 mM ethylene glycol tetraacetic acid, 1 mM MgSO_4_, 3.7% formaldehyde). The late larvae and juveniles were perfused transcardially with 0.9% sodium chloride, followed by the same fixative solutions. The brains were dissected out and postfixed for 3 hours at 4°C.

Maturational aspects in the developing *X. laevis* retina were examined in semithin (morphological analysis) and cryostat sections (immunohistochemical analysis). For the morphological analysis, some fixed embryos and postnatal specimens were rinsed in PB, postfixed in 2% osmium tetroxide for 2 h, dehydrated in a graded series of acetone and propylene oxide, and embedded in Spurr's resin. Serial frontal 2 *μ*m sections were cut in a Reichert Jung microtome. The sections were stained with 1% toluidine blue in 1% aqueous borax.

For immunohistochemical analysis, tissues were rinsed in PB, then cryoprotected, soaked in embedding medium, frozen, and freeze-mounted onto aluminium sectioning blocks. Cryostat sections, 15 *μ*m thick, were cut in the frontal plane. Sections through different retinal areas were thaw-mounted on SuperFrost Plus slides (Menzel-Glaser, Germany), air-dried, and stored at −80°C.

### 2.2. Immunohistochemistry

Working solutions and sources of primary and secondary antibodies used in the present study are summarized in Table 2. The anti-Isl1 monoclonal antibody [38] was developed by Dr. Jessell (Columbia University). It has recently been used and tested in mouse, chicken, turtle, and *Xenopus*, with comparable results [12, 18, 31, 33, 34, 39] (see also data sheet DSHB), and the staining pattern colocalized with the mRNA distribution [40]. The rest of the primary antibodies used in this report have been widely used in neuroanatomical studies in the central nervous system of different groups of vertebrates. They cross react with antigens present in the *X*. *laevis* tissue, showing similar staining patterns to those previously described in other vertebrate retinas. Single- and double-immunohistochemical studies were performed as described in Bejarano-Escobar, Blasco [9] and simultaneously labeled with the nuclear dye 4′,6-diamino-2-phenylindole (DAPI). Sections were coverslipped with Mowiol for observation. Controls for the Isl1 immunostaining included (i) Western blot for the Isl1 antibody which showed a band in *Xenopus*, *Rana*, and rat that corresponded to the Isl1 LIM homeobox-1 because it coincides with the expected molecular weight (about 39 kDa); [41]; (ii) incubation of some selected sections with preimmune mouse or rabbit sera (1 : 1000 dilution) instead of the primary antibody; (iii) controls in which either the primary or the secondary antibody was omitted; and (iv) preadsorption of the primary antibody with synthetic peptide (Isl1 peptide, Abcam, Cambridge, MA, USA) [42]. In all the latter controls, the immunostaining was omitted. Controls for the other antibodies used in combination with Isl1 can be found elsewhere: CB, CR [43], CERN-922 [9–11], and SV2 [10, 11].

### 2.3. Image Acquisition and Processing

Digital images of *X. laevis* specimens were captured with a Digital Camera DS-5Mc (Nikon) attached to a Stereoscopic Microscope SMZ-1000 (Nikon). Immunolabeled sections were observed using an epifluorescence, bright field Nikon Eclipse 80i microscope, and photographed using an ultra-high-definition Nikon digital camera DXM1200F. Some of the double-immunolabelings were photographed with a Nikon D-Eclipse C1 confocal laser-scanning microscope. Graphical enhancement and preparation for publication were performed in Adobe Photoshop (v.CS4).

## 3. Results

All the retinal cell types during development of *Xenopus laevis* are generated within 36 hours (between St24 and St40) after the birth of the first retinal ganglion cells [44]. Therefore, the retinal expression of Isl1 was examined carefully from the early embryonic St24 through the juvenile period. No Isl1-immunoreactive elements were distinguishable before St29-30. From the onset of the appearance of Isl1 expression in the retina (St29-30), immunoreactivity appeared progressively following central-to-peripheral and vitreal-to-scleral gradients. From St42 onwards all retinal cell types were fully differentiated and the Isl1 expression pattern did not change significantly.

### 3.1. Isl1 Expression in the *X. laevis* Differentiated Retina

**Figure 2:**
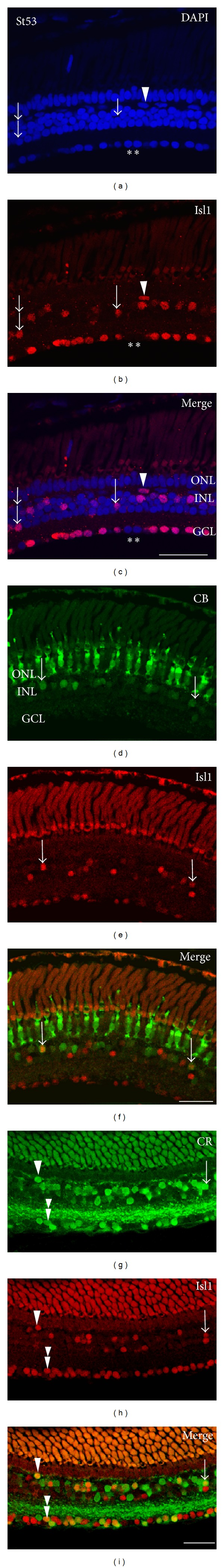
Morphological features and expression patterns of Isl1 and other cell differentiation markers in the St53 *Xenopus laevis* central retina. (a)–(c) DAPI fluorescence combined with Isl1 immunofluorescence. DAPI staining showed well-organized retinal layers. Isl1 expressing cells in the INL could be characterized morphologically and topographically as horizontal (arrowheads), bipolar (arrows), or amacrine cells (double arrows). Notice that not all cells located in the GCL expressed Isl1 (asterisks). (d)–(f) A few Isl1-positive bipolar cells also expressed CB (arrows). (g)–(i) Subpopulations of Isl1-positive horizontal (arrowheads), bipolar (arrows), and ganglion cells (double arrowheads) also expressed CR. GCL: ganglion cell layer; INL: inner nuclear layer; ONL: outer nuclear layer. Scale bars: 100 *μ*m.

The Isl1 expression pattern in fully differentiated retinas will be reported first so as to establish a set of referents with which to compare developmental stages. Thus, at St53, the retina is almost fully developed, exhibiting complete lamination as determined from DAPI-stained cryosections (Figure 2(a)). Anti-Isl1 antibody stained most of the cells located in the ganglion cell layer (GCL) (Figures 2(b), 2(c), 2(e), 2(f), 2(h), and 2(i)). Furthermore, different populations of Isl1-containing cells located in the inner nuclear layer (INL) could be distinguished by both morphological and topographical features: (i) a small population of cells with the major axis oriented parallel to the vitreal surface, lying at the border between the outer plexiform layer (OPL) and the INL, in the horizontal cell layer (Figures 2(b) and 2(c)); (ii) a population of cells located in the middle of the INL, in the bipolar cell layer (Figures 2(b), 2(c), 2(e), 2(f), 2(h), and 2(i)); and (iii) a small population of cells located at the inner edge of the INL, in the region where the amacrine cells are normally found (Figures 2(b), 2(c), 2(e), 2(f), 2(h), and 2(i)). To further characterize Isl1-expressing cells, a double labeling with antibodies against calbindin-D28k (CB) and calretinin (CR) was also performed. In the developing and adult *X. laevis* retina, CB labels cones and subpopulations of bipolar, amacrine, and ganglion cells [35, 45]. Some of the CB-immunoreactive bipolar cells also expressed Isl1 (Figures 2(d)–2(f)). CR labels subsets of horizontal, bipolar, amacrine, and ganglion cells [35]. Some of the CR-expressing horizontal, bipolar, and ganglion cells also expressed Isl1 (Figures 2(g)–2(i)).

### 3.2. Isl1 Expression in the *X. laevis* Developing Retina

**Figure 3:**
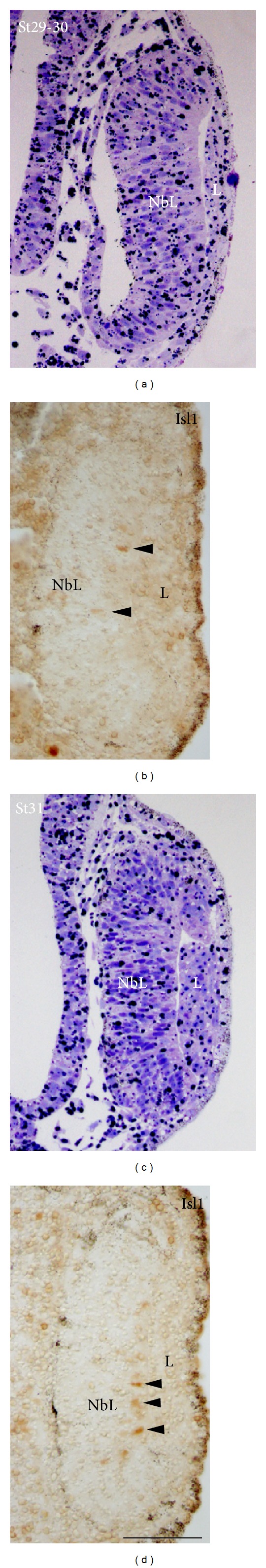
Morphological features and expression patterns of Isl1 in the St29/30 ((a), (b)) and St31 ((c), that Isl1 is also expressed by undi (d)) *Xenopus laevis* retina. ((a), (c)) Toluidine blue-stained transverse retinal resin sections showed that the neural retina had the structure of pseudostratified columnar epithelium. Notice that during these early stages abundant dark-stained granules were observed in the cytoplasm of the neuroepithelial cells. (b),(d) Isl1 immunoreactivity was present in sparse nuclei mainly located near the vitreal surface (arrowheads). L: lens; NbL: neuroblastic layer. Scale bar: 50 *μ*m.

**Figure 4:**
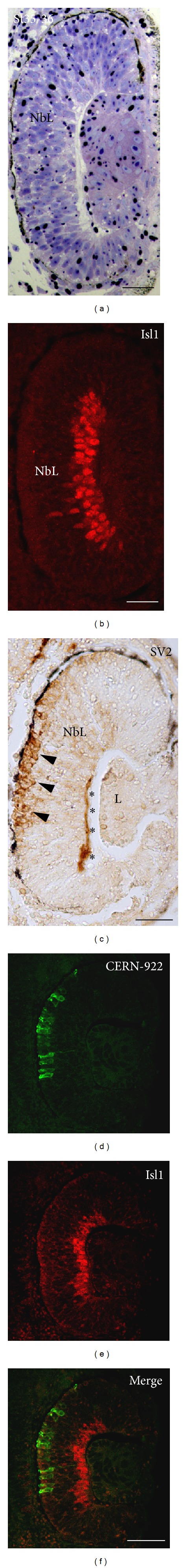
Morphological features and expression patterns of Isl1 and other cell differentiation markers in the St35/36 *Xenopus laevis* retina. (a) Toluidine blue-stained transverse retinal resin sections showed that neural retina remained undifferentiated during this stage. (b) Although no plexiform layers were observed, many Isl1-positive nuclei were mainly located near the inner surface of the neuroretina. (c) Immunoreactive ganglion cell axons for SV2 were observed in the vitreal surface of the retina (asterisks). Furthermore, immunoreactivity was also detected near the scleral surface of the undifferentiated retina (arrowheads). (d)–(f) CERN-922 antibody revealed that sparse photoreceptors were differentiating by this stage in the scleral surface of the central nonlayered retina ((d), (F)). L: lens; NbL: neuroblastic layer. Scale bars: 50 *μ*m.

By St29-30, although the lumen of the ventricle was still observed, the most distal part of the vesicle wall had invaginated to form a two-layered optic cup (Figure 3(a)). The neural retina was composed of a neuroblastic layer (NbL) (Figure 3(a)), and weak Isl1 immunoreactivity was first observed in sparse nuclei located near the vitreal surface of the central retina (Figure 3(b)). Similar morphological features and staining patterns were observed at St31 (Figures 3(c) and 3(d)). The number of Isl1-positive cells progressively increased, and at St35/36, albeit layering was still observed, the retina was losing its pseudostratified, undifferentiated appearance (Figure 4(a)). At these stages, Isl1 expression was detected in abundant newly formed postmitotic cells that accumulated in the vitreal surface of the central and mid-peripheral undifferentiated retina and in sparse migrating neuroblasts in outer regions of the NbL (Figure 4(b)). We compared the Isl1 expression pattern with that of other cell differentiation markers. Thus, we used an antibody against the transmembrane synaptic vesicle glycoprotein SV2 that has been demonstrated to be a powerful tool to label the first optic axons emerging from young ganglion cells [10, 11, 46] and also to address the appearance of functional synapses [10, 11, 46–49]. Thus, strong SV2-immunoreactive ganglion cell axons were detected in the presumptive optic fibre layer (OFL), coursing to the optic nerve exit (Figure 4(c)). Surprisingly, strong SV2 immunoreactivity was also found in the scleralmost part of the neuroepithelium, suggesting that differentiating cells were also located in this region (Figure 4(c)). Therefore, we used a polyclonal antibody against bovine rod opsin (CERN-922) that has been described as an excellent photoreceptor marker in fish [9–11, 50–53], reptiles (unpublished observations), birds (unpublished observations), and mammals [54]. A similar antibody has been previously used to identify rod photoreceptors in the developing *X. laevis* retina [45]. Sparse morphologically immature CERN-922-immunoreactive photoreceptors were detected in the central region of the retina (Figures 4(d)–4(f)). Therefore, in discordance with the undifferentiated morphological appearance of the *X. laevis* retina, several differentiating cell populations could be observed by these stages.

**Figure 5:**
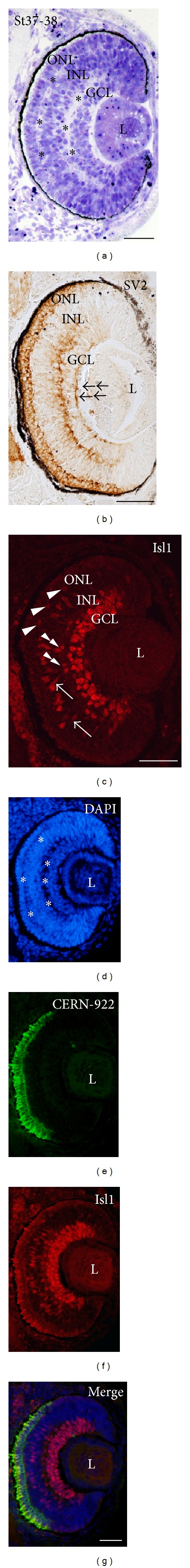
Morphological features and expression patterns of Isl1 and other cell differentiation markers in the St37/38 *Xenopus laevis* retina. (a) Toluidine blue-stained transverse retinal resin sections showed that the plexiform layers were observed across the central to mid-peripheral extent of the retina (asterisks). An immature GCL, 2-3 cells in depth, was also observed at this stage. (b) SV2 immunoproducts were observed in the OFL (double arrows), IPL, and ONL. (c) Abundant nuclei were immunoreactive for Isl1 in the GCL, but also in the INL. Thus, nuclei located in the amacrine cell layer (double arrowheads), bipolar cell layer (arrows), and horizontal cell layer (arrowheads) were detected with this antibody. (d)–(g) Incipient plexiform layers were also clearly distinguishable with the DAPI nucleic acid stain (asterisks in (d)). CERN-922 immunoreactivity paralleled the expression of Isl1 and extended towards the more mid-peripheral region of the retina. GCL: ganglion cell layer; INL: inner nuclear layer; L: lens; ONL: outer nuclear layer. Scale bars: 50 *μ*m.

At St 37/38, the inner plexiform layer (IPL) was formed above the GCL, which was several cells thick, along the middle third of the retina (Figures 5(a), 5(d), and 5(g)). Furthermore, at this stage also, the first sign of the OPL could be observed, separating the outer nuclear layer (ONL) from the INL (Figures 5(a), 5(d), and 5(g)). SV2 antibody also revealed the emergence of the plexiform layers in the central and the mid-peripheral retina (Figure 5(b)). By this stage, most Isl1-immunoreactive cells were confined to the newly formed GCL (Figure 5(c)). However, many Isl1-positive cells appeared at this stage in the INL. On the basis of laminar position and morphology, these cells could be identified as horizontal, bipolar, and amacrine cells (Figure 5(c)). Isl1 and CERN-922 immunoreactivities progressed in parallel following a central-to-peripheral gradient, but Isl1 immunoreactivity extended to more peripheral regions (Figures 5(d)–5(g)).

**Figure 6:**
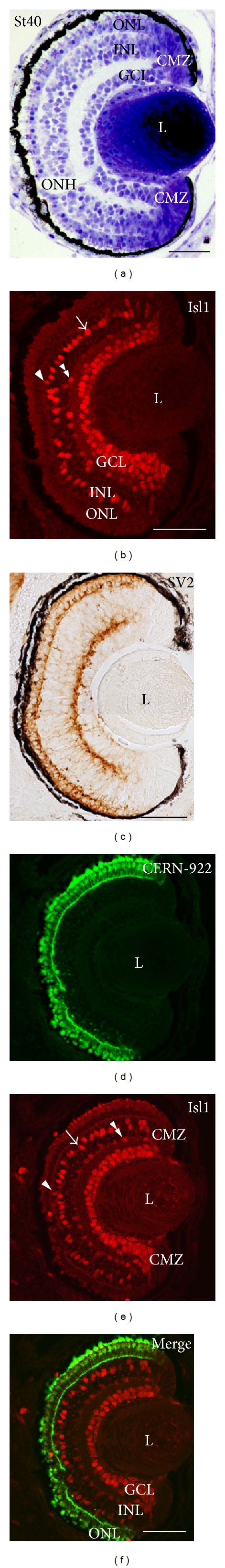
Morphological features and expression patterns of Isl1 and other cell differentiation markers in the St40 *Xenopus laevis* retina. (a) Toluidine blue-stained transverse retinal resin sections revealed that the typical multilayered structure was clearly distinguishable, showing morphological features of maturation. The CMZ was visible in the peripheralmost region. (b) Isl1 immunoreactivity was clearly established in abundant nuclei located in the GCL, whereas the INL contained Isl1 labeling that corresponded to horizontal (arrowhead), bipolar (arrow), and amacrine cells (double arrowhead). (c) SV2 expression in the plexiform layers extended to more peripheral regions. (d)–(f) The CERN-922 labeling in the ONL coursed in parallel with Isl1 immunoreactivity and reached the periphery by this stage. CMZ: ciliary marginal zone; GCL: ganglion cell layer; INL: inner nuclear layer; L: lens; ONH: optic nerve head; ONL: outer nuclear layer. Scale bars: 100 *μ*m.

**Figure 7:**
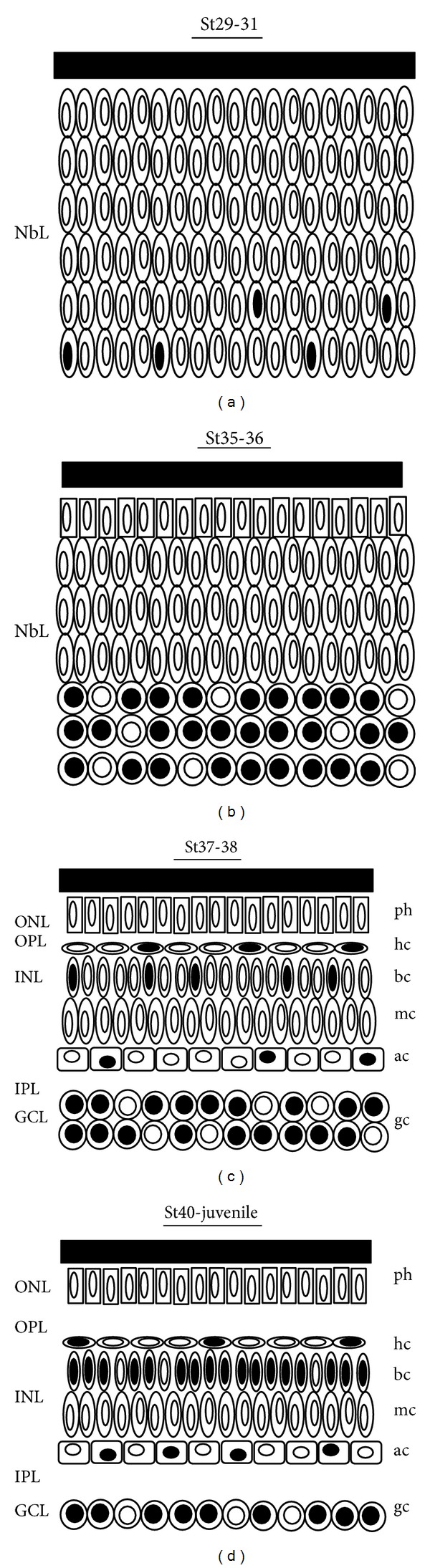
Schematic drawings of the Isl1 positive cells in the developing retina of *Xenopus laevis*. Black nuclei represent Isl1-immunostained cells. (a) Isl1 immunoreactivity during St29–31 was restricted to nuclei located near the vitreal surface of the NbL. (b) Although the retinal lamination was absent in the St35-36, cell differentiation was detected immunohistochemically. Abundant Isl1-positive cells were located in the vitreal half of the NbL. (c) At St37-38, retinal layering was completed in the central retina with the emergence of the plexiform layers. Isl1 immunoreactivity was detected in the GCL and in cell subpopulations located in the INL. (d) From St40 onwards Isl1 immunoreactivity was confined to subpopulations of ganglion, amacrine, bipolar, and horizontal cells. Ac: amacrine cell; bc: bipolar cell; gc: ganglion cell; GCL: ganglion cell layer; hc: horizontal cell; INL: inner nuclear layer; IPL: inner plexiform layer; L: lens; mc: Müller cell; NbL: neuroblastic layer; ONL: outer nuclear layer; OPL: outer plexiform layer; ph: photoreceptor.

At St40, the typical multilayered structure of the retina was clearly distinguishable. However, the GCL was many cells thick, and the plexiform layers were poorly developed (Figure 6(a)). A proliferative population of undifferentiated cells remained at the ciliary margin zone (CMZ), providing a source of new cells for the growing retina (Figure 6(a)). At this stage, the expression patterns for Isl1 (Figures 6(b), 6(e), and 6(f)), SV2 (Figure 6(c)), and CERN-922 (Figures 6(d) and 6(f)) followed a central-to-peripheral gradient. The number of Isl1 immunoreactive elements and the immunostaining intensity increased markedly in the INL and began to resemble those observed in the St53 retina (Figure 2). The patterns of distribution of Isl1 positive cells during *X. laevis* retinal development are summarized in Figure 7.

## 4. Discussion

Recent studies conducted in our laboratory confirmed the presence of the LIM-domain transcription factor Isl1 in differentiating and mature ganglion, amacrine, bipolar, and horizontal cells in the retina of mammals [54], birds [18], reptiles [12], and fish [9–11]. In the present study, we extend our observations to the retina of the anuran *Xenopus laevis*, an amphibian model extensively used in studies of retinal cell organization [44, 55–57]. We first established the precise cell types that showed immunoreactivity for Isl1 in the differentiated retina, and subsequently analyzed the Isl1 immunoreactivity in the corresponding cells in the retina throughout development. The Isl1 spatiotemporal expression pattern spans the differentiation of four neuronal classes—ganglion, amacrine, bipolar, and horizontal cells—in the developing *X. laevis* retina.

### 4.1. Isl1 Expression in the Nonlayered Retina of *X. laevis*

Between St29 and 30, the first detectable Isl1 expression, and St 35-36, the plexiform layers were not recognizable in the developing *X. laevis* retina. By these stages, the distribution of Isl1 expression was consistent with that expected for a transcription factor involved in retinal neuroblast differentiation. Indeed, antibodies against Isl1 have been used in retinal studies of cell neurogenesis, migration, and early differentiation in the developing retina of different vertebrates [9–12, 15–17, 22, 23, 25–27]. Studies on cell birthdays in *X. laevis* retina have shown that the first retinal ganglion cells are born at St24-25, approximately 26 hours after fertilization [44, 58] and that the pioneer ganglion cell axons appear in the retina at St28 [55]. Isl1 expression was first detected at St29/30 (approximately 7 h after the first ganglion cells are born) in ovoid nuclei of apparently migrating neuroblasts dispersed throughout the NbL. Therefore, the expression of Isl1 in the developing *X. laevis* retina starts at slightly later stages than that of the onset of ganglion cell neurogenesis but coincides with early stages of ganglion cell differentiation.

In more advanced stages (St35-36), although still no layering is observed, the *X. laevis* retina progressively loses its pseudostratified, undifferentiated appearance, most ganglion cell genesis is completed [59, 60], and several distinct differentiating cell populations can be identified neurochemically [45, 58, 61, 62] (present study). Isl1-immunoreactive cell nuclei were mostly located towards the inner side of the retina where the GCL is forming but also in newly formed migratory neuroblasts still located in the NbL. However, Isl1 expression is greater in the presumptive GCL than in the NbL, as has also been observed in fish [11], reptiles [12], birds [16–18], and mammals [22, 26]. Since Isl1 is known to be expressed by mature and differentiating ganglion cells, many of the immunoreactive cells located vitreally may correspond to this cell type. However, it has been reported that Isl1 is also present in undifferentiated amacrine, bipolar and horizontal cells in the retina of vertebrates [9–12, 16, 17, 22, 23, 26]. In particular, Isl1 controls the differentiation of cholinergic amacrine cells and also may function in the specification of bipolar cell subtype or the differentiation of previously specified bipolar subtypes in the murine retina [22, 23]. Furthermore, Isl1 immunostaining has been used to determine the onset of differentiation of horizontal cells in the avian retina [16, 63]. Therefore, we cannot be sure that all Isl1-immunoreactive cells labeled by these early stages were mature or differentiating ganglion cells. All these data suggest that Isl1 seems to be a reliable marker for newly generated neurons in the *X. laevis* retina.

### 4.2. Isl1 Expression in the Layered Retina of *X. laevis*

Changes in the expression pattern of Isl1 in the *X. laevis* retina became apparent at St37/38. By these stages, ganglion cells extend short unbranched primary dendrites within the nascent IPL [56, 64], and the retina becomes organized into three nuclear layers: GCL, INL, and ONL [57]. Regarding the Isl1 expression in the GCL in different species, it has been identified in a predominant fraction of retinal ganglion cell nuclei [9–13, 17, 24–26, 32, 33]. Some of the Isl1-immunoreactive ganglion cells also expressed other typical ganglion cell markers, such as CR, as has previously been described in other vertebrates [9, 10].

With respect to the INL, most Isl1-expressing cells were detected along the outermost border of the INL, where bipolar and horizontal cells reside. In addition, scattered Isl1-positive cells are located along the inner border of the INL, in the amacrine cell layer. In other species, the orderly array of scattered Isl1-positive cells along the innermost region of the INL has been shown to include a mosaic of cholinergic amacrine cells [9–12, 17, 22, 24]. Isl1 expression was also detected in bipolar cells that also expressed typical bipolar cell markers such as CR or CB, as has previously been shown in the fish retina [9, 10]. Finally, we also found Isl1 expression in subsets of differentiated horizontal cells, in coherence with the results described previously in the retina of fish [10, 11], reptiles [12], and birds [14–17]. However, Isl1 is not expressed by horizontal cells in the developing and adult retina of mammals [22, 23].

In conclusion, the expression of Isl1 in subsets of mature and differentiating ganglion, amacrine, and bipolar cells is consistent across species from fish to mammals, supporting the hypothesis that it has an essential role in vertebrate retinal cell specification, differentiation, and maintenance.

## Figures and Tables

**Table 1:** Number of animals investigated at different stages of development with Isl1 immunohistochemistry.

Developmental stages*						
Embryonic		Premetamorphic	Prometamorphic	Metamorphic climax	Juveniles	*n*
Early	Late					

24–37	38–45	46–51	53–59	60–66

30	30	6	7	7	2	82

*Staging of the *Xenopus laevis* embryos and larvae according to Nieuwkoop and Faber, 1967 [37].

**Table 2:** Immunoreagents, working dilutions, and sources of antibodies used in the present study.

	Working dilution	Antibody suppliers (reference)
Primary antibody		
Rabbit anti-bovine rod opsin polyclonal antibody, CERN-922	1 : 1000	Gift from Dr. Willem J. DeGrip
Mouse anti-Islet-1 monoclonal antibody (clone 39.4D5)	1 : 200	Developmental Studies Hybridoma Bank (DSHB)
Mouse anti-SV2 monoclonal antibody	1 : 200	DSHB
Rabbit anti-calbindin polyclonal antibody	1 : 1000	Swant (Ref. CB-38a)
Rabbit anti-calretinin polyclonal antibody	1 : 2000	Swant (Ref. 7699-4)
Secondary antibodies		
Alexa Fluor 594 goat-anti-mouse IgG antibody	1 : 200	Molecular Probes, The Netherlands
Alexa Fluor 488 goat-anti-rabbit IgG antibody	1 : 200	Molecular Probes, The Netherlands
Anti-mouse IgG biotin conjugate	1 : 200	Sigma (B 7264)

## References

[1] Young RW (1985). Cell proliferation during postnatal development of the retina in the mouse. *Brain Research*.

[2] Cepko CL (1996). The patterning and onset of opsin expression in vertebrate retinae. *Current Opinion in Neurobiology*.

[3] Marquardt T, Ashery-Padan R, Andrejewski N, Scardigli R, Guillemot F, Gruss P (2001). Pax6 is required for the multipotent state of retinal progenitor cells. *Cell*.

[4] Wang SW, Kim BS, Ding K (2001). Requirement for math5 in the development of retinal ganglion cells. *Genes and Development*.

[5] Cheng CW, Chow RL, Lebel M (2005). The Iroquois homeobox gene, Irx5, is required for retinal cone bipolar cell development. *Developmental Biology*.

[6] Chow RL, Volgyi B, Szilard RK (2004). Control of late off-center cone bipolar cell differentiation and visual signaling by the homeobox gene Vsx1. *Proceedings of the National Academy of Sciences of the United States of America*.

[7] Pfaff SL, Mendelsohn M, Stewart CL, Edlund T, Jessell TM (1996). Requirement for LIM homeobox gene Isl1 in motor neuron generation reveals a motor neuron-dependent step in interneuron differentiation. *Cell*.

[8] Hobert O, Westphal H (2000). Functions of LIM-homeobox genes. *Trends in Genetics*.

[9] Bejarano-Escobar R, Blasco M, DeGrip WJ, Martín-Partido G, Francisco-Morcillo J (2009). Cell differentiation in the retina of an epibenthonic teleost, the Tench (*Tinca tinca*, Linneo 1758). *Experimental Eye Research*.

[10] Bejarano-Escobar R, Blasco M, DeGrip WJ, Oyola-Velasco JA, Martín-Partido G, Francisco-Morcillo J (2010). Eye development and retinal differentiation in an altricial fish species, the senegalese sole (*Solea senegalensis*, Kaup 1858). *Journal of Experimental Zoology Part B*.

[11] Bejarano-Escobar R, Blasco M, Durán AC, Rodríguez C, Martín-Partido G, Francisco-Morcillo J (2012). Retinal histogenesis and cell differentiation in an elasmobranch species, the small-spotted catshark *Scyliorhinus canicula*. *Journal of Anatomy*.

[12] Francisco-Morcillo J, Hidalgo-Sánchez M, Martín-Partido G (2006). Spatial and temporal patterns of proliferation and differentiation in the developing turtle eye. *Brain Research*.

[13] Fischer AJ, Dierks BD, Reh TA (2002). Exogenous growth factors induce the production of ganglion cells at the retinal margin. *Development*.

[14] Fischer AJ, Stanke JJ, Aloisio G, Hoy H, Stell WK (2007). Heterogeneity of horizontal cells in the chicken retina. *Journal of Comparative Neurology*.

[15] Boije H, Edqvist P-HD, Hallböök F (2008). Temporal and spatial expression of transcription factors FoxN4, Ptf1a, Prox1, Isl1 and Lim1 mRNA in the developing chick retina. *Gene Expression Patterns*.

[16] Edqvist P-HD, Lek M, Boije H, Lindbäck SM, Hallböök F (2008). Axon-bearing and axon-less horizontal cell subtypes are generated consecutively during chick retinal development from progenitors that are sensitive to follistatin. *BMC Developmental Biology*.

[17] Edqvist PHD, Myers SM, Hallböök F (2006). Early identification of retinal subtypes in the developing, pre-laminated chick retina using the transcription factors Prox1, Lim1, Ap2*α*, Pax6, Isl1, Isl2, Lim3 and Chx10. *European Journal of Histochemistry*.

[18] Francisco-Morcillo J, Sánchez-Calderón H, Kawakami Y, Izpisúa-Belmonte JC, Hidalgo-Sánchez M, Martín-Partido G (2005). Expression of Fgf19 in the developing chick eye. *Brain Research Developmental Brain Research*.

[19] Suga A, Taira M, Nakagawa S (2009). LIM family transcription factors regulate the subtype-specific morphogenesis of retinal horizontal cells at post-migratory stages. *Developmental Biology*.

[20] Okamoto M, Bito T, Noji S, Ohuchi H (2009). Subtype-specific expression of Fgf19 during horizontal cell development of the chicken retina. *Gene Expression Patterns*.

[21] Austin CP, Feldman DE, Ida JA, Cepko CL (1995). Vertebrate retinal ganglion cells are selected from competent progenitors by the action of Notch. *Development*.

[22] Elshatory Y, Deng M, Xie X, Gan L (2007). Expression of the LIM-homeodomain protein Isl1 in the developing and mature mouse retina. *Journal of Comparative Neurology*.

[23] Elshatory Y, Everhart D, Deng M, Xie X, Barlow RB, Gan L (2007). Islet-1 controls the differentiation of retinal bipolar and cholinergic amacrine cells. *Journal of Neuroscience*.

[24] Galli-Resta L, Resta G, Tan S-S, Reese BE (1997). Mosaics of Islet-1-expressing amacrine cells assembled by short-range cellular interactions. *Journal of Neuroscience*.

[25] Mu X, Fu X, Beremand PD, Thomas TL, Klein WH (2008). Gene-regulation logic in retinal ganglion cell development: Isl1 defines a critical branch distinct from but overlapping with Pou4f2. *Proceedings of the National Academy of Sciences of the United States of America*.

[26] Pan L, Deng M, Xie X, Gan L (2008). ISL1 and BRN3B co-regulate the differentiation of murine retinal ganglion cells. *Development*.

[27] Wu F, Sapkota D, Li R, Mu X (2012). Onecut 1 and Onecut 2 are potential regulators of mouse retinal development. *Journal of Comparative Neurology*.

[28] Kiyama T, Mao C-A, Cho J-H (2011). Overlapping spatiotemporal patterns of regulatory gene expression are required for neuronal progenitors to specify retinal ganglion cell fate. *Vision Research*.

[29] Stanke JJ, Lehman B, Fischer AJ (2008). Muscarinic signaling influences the patterning and phenotype of cholinergic amacrine cells in the developing chick retina. *BMC Developmental Biology*.

[30] Whitney IE, Raven MA, Ciobanu DC (2011). Genetic modulation of horizontal cell number in the mouse retina. *Proceedings of the National Academy of Sciences of the United States of America*.

[31] Moreno N, Domínguez L, Retaux S, González A (2008). Islet1 as a marker of subdivisions and cell types in the developing forebrain of Xenopus. *Neuroscience*.

[32] Dorsky RI, Chang WS, Rapaport DH, Harris WA (1997). Regulation of neuronal diversity in the *Xenopus* retina by delta signalling. *Nature*.

[33] Ma L, Hocking JC, Hehr CL, Schuurmans C, McFarlane S (2007). Zac1 promotes a Müller glial cell fate and interferes with retinal ganglion cell differentiation in *Xenopus* retina. *Developmental Dynamics*.

[34] Bilitou A, De Marco N, Bello AM (2012). Spatial and temporal expressions of prune reveal a role in Muller gliogenesis during *Xenopus* retinal development. *Gene*.

[35] Morona R, Moreno N, López JM, González A (2007). Comparative analysis of calbindin D-28K and calretinin in the retina of anuran and urodele amphibians: colocalization with choline acetyltransferase and tyrosine hydroxylase. *Brain Research*.

[36] Morona R, Northcutt RG, González A (2011). Immunohistochemical localization of calbindin D28k and calretinin in the retina of two lungfishes, *Protopterus dolloi* and *Neoceratodus forsteri*: colocalization with choline acetyltransferase and tyrosine hydroxylase. *Brain Research*.

[37] Nieuwkoop PD, Faber J (1967). *Normal Table of Xenopus Laevis (Daudin)*.

[38] Ericson J, Thor S, Edlund T, Jessell TM, Yamada T (1992). Early stages of motor neuron differentiation revealed by expression of homeobox gene Islet-1. *Science*.

[39] Moreno N, Morona R, López JM, González A (2010). Subdivisions of the turtleseudemys scripta subpallium based on the expression of regulatory genes and neuronal markers. *Journal of Comparative Neurology*.

[40] Wang H-F, Liu F-C (2001). Developmental restriction of the LIM homeodomain transcription factor Islet-1 expression to cholinergic neurons in the rat striatum. *Neuroscience*.

[41] Moreno N, Morona R, López JM (2012). Characterization of the bed nucleus of the stria terminalis in the forebrain of anuran amphibians. *Journal of Comparative Neurology*.

[42] Moreno N, González A, Rétaux S (2008). Evidences for tangential migrations in *Xenopus* telencephalon: developmental patterns and cell tracking experiments. *Developmental Neurobiology*.

[43] Morona R, Moreno N, Lopez JM, Muñoz M, Domínguez L, González A (2008). Calbindin-D28k and calretinin as markers of retinal neurons in the anuran amphibian *Rana perezi*. *Brain Research Bulletin*.

[44] Holt CE, Bertsch TW, Ellis HM, Harris WA (1988). Cellular determination in the xenopus retina is independent of lineage and birth date. *Neuron*.

[45] Chang WS, Harris WA (1998). Sequential genesis and determination of cone and rod photoreceptors in *Xenopus*. *Journal of Neurobiology*.

[46] Bergmann M, Grabs D, Rager G (1999). Developmental expression of dynamin in the chick retinotectal system. *Journal of Histochemistry and Cytochemistry*.

[47] Okada M, Erickson A, Hendrickson A (1994). Light and electron microscopic analysis of synaptic development in Macaca monkey retina as detected by immunocytochemical labeling for the synaptic vesicle protein, SV2. *Journal of Comparative Neurology*.

[48] Misgeld T, Burgess RW, Lewis RM, Cunningham JM, Lichtman JW, Sanes JR (2002). Roles of neurotransmitter in synapse formation: development of neuromuscular junctions lacking choline acetyltransferase. *Neuron*.

[49] Blanchart A, Romaguera M, García-Verdugo JM, De Carlos JA, López-Mascaraque L (2008). Synaptogenesis in the mouse olfactory bulb during glomerulus development. *European Journal of Neuroscience*.

[50] Meléndez-Ferro M, Villar-Cheda B, Abalo XM (2002). Early development of the retina and pineal complex in the sea lamprey: comparative immunocytochemical study. *Journal of Comparative Neurology*.

[51] Candal E, Anadón R, Degrip WJ, Rodríguez-Moldes I (2005). Patterns of cell proliferation and cell death in the developing retina and optic tectum of the brown trout. *Developmental Brain Research*.

[52] Ferreiro-Galve S, Rodríguez-Moldes I, Anadón R, Candal E (2010). Patterns of cell proliferation and rod photoreceptor differentiation in shark retinas. *Journal of Chemical Neuroanatomy*.

[53] Bejarano-Escobar R, Blasco M, Martín-Partido G, Francisco-Morcillo J (2013). Light-induced degeneration and microglial response in the retina of an epibenthonic pigmented teleost: age-dependent photoreceptor susceptibility to cell death. *The Journal of Experimental Biology*.

[54] Bejarano-Escobar R, Holguín-Arévalo MS, Montero JA, Francisco-Morcillo J, Martín-Partido G (2011). Macrophage and microglia ontogeny in the mouse visual system can be traced by the expression of Cathepsins B and D. *Developmental Dynamics*.

[55] Cima C, Grant P (1980). Ontogeny of the retina and optic nerve of *Xenopus laevis*. IV. Ultrastructural evidence of early ganglion cell differentiation. *Developmental Biology*.

[56] Holt CE (1989). A single-cell analysis of early retinal ganglion cell differentiation in *Xenopus*: from soma to axon tip. *Journal of Neuroscience*.

[57] Chung SH, Stirling RV, Gaze RM (1975). The structural and functional development of the retina in larval *Xenopus*. *Journal of Embryology and Experimental Morphology*.

[58] Stiemke MM, Hollyfield JG (1995). Cell birthdays in *Xenopus laevis* retina. *Differentiation*.

[59] Hirsch N, Harris WA (1997). Xenopus Brn-3.0, a POU-domain gene expressed in the developing retina and tectum: not regulated by innervation. *Investigative Ophthalmology and Visual Science*.

[60] Hirsch N, Harris WA (1997). *Xenopus* Pax-6 and retinal development. *Journal of Neurobiology*.

[61] Rapaport DH, Patheal SL, Harris WA (2001). Cellular competence plays a role in photoreceptor differentiation in the developing *Xenopus* retina. *Journal of Neurobiology*.

[62] Hocking JC, McFarlane S (2007). Expression of Bmp ligands and receptors in the developing *Xenopus* retina. *International Journal of Developmental Biology*.

[63] Boije H, Edqvist P-HD, Hallböök F (2009). Horizontal cell progenitors arrest in G2-phase and undergo terminal mitosis on the vitreal side of the chick retina. *Developmental Biology*.

[64] Lom B, Cogen J, Sanchez AL, Vu T, Cohen-Cory S (2002). Local and target-derived brain-derived neurotrophic factor exert opposing effects on the dendritic arborization of retinal ganglion cells in vivo. *Journal of Neuroscience*.

